# Periprocedural Hemoglobin Changes and Myocardial Injury in Patients Undergoing Percutaneous Coronary Intervention

**DOI:** 10.5812/cardiovascmed.9595

**Published:** 2013-07-31

**Authors:** Keivan Alizadeh, Saloomeh Pouralizadeh, Sepideh Parchami-Ghazaee, Abbas Zavvarehee, Seifollah Abdi, Farshad Shakerian, Behshid Ghadrdoost, Shiva Khaleghparast, Negar Salehi, Ata Firouzi, Mohsen Maadani

**Affiliations:** 1 Cardiovascular Intervention Research Center, Rajaie Cardiovascular Medical and Research Center, Iran University of Medical Sciences, Tehran, IR Iran

**Keywords:** Angioplasty, Hemoglobin, Anemia

## Abstract

**Background:**

Patients undergoing percutaneous coronary intervention are at serious risk of different complications such as periprocedural bleeding that can lead to myocardial injuries. Blood loss through puncture site hematoma formation and through catheter aspiration causes periprocedural hemoglobin drop.

**Objectives:**

Although percutaneous coronary intervention is an effective treatment of coronary artery disease, it seems necessary to investigate the impact of complications on outcomes including myocardial infarction and possible mortality. The purpose of this study was to evaluate the relationship between periprocedural hemoglobin levels and cardiac enzyme changes as a predictor of cardiac adverse outcomes in patients undergoing percutaneous coronary intervention.

**Patients and Methods:**

This study was conducted on 1012 consecutive patients with a diagnosis of coronary artery disease who underwent percutaneous coronary intervention. Hemoglobin levels were measured immediately before and post-procedurally and based on the baseline levels the patients were classified into anemic and non-anemic groups. The samples for TnI and CP-MB were collected before the procedure and at 8, 16, and 24 hours post-procedurally. The patients were stratified into three categories of myocardial injuries: patients with CK-MB ≥ 3×; those with TnI > 0.06 µu/l and individuals with both CK-MB ≥ 3× and TnI > 0.06 µu/l.

**Results:**

All categories divided by cardiac enzyme status either in positive or in negative groups were classified in non-anemic group. Although in all groups hemoglobin level decreased post-procedurally, in the second category (TnI ≥ 0.06) the positive patients had significantly lower hemoglobin amounts after the procedure (P = 0.008).

**Conclusion:**

Post-procedure hemoglobin dropping may be considered as a predictor of cardiac adverse outcome in patients undergoing PCI. We suggest that a good bleeding control during and after the procedure can reduce the risk of cardiac enzyme elevation post-procedurally.

## 1. Background

Patients undergoing percutaneous coronary intervention (PCI) are at a serious risk of different complications that can lead to myocardial injuries. Periprocedural bleeding is one of the most common complications of PCI (1). These myocardial injury related factors are categorized as patient-related, lesion-related and procedure-related (2). Older age, female sex, lower body weight, low cholesterol, multi-lesion intervention and heparin therapy independently correlate with increased risk of bleeding in patients enrolled in intracoronary stenting (3). Blood loss through puncture site hematoma formation and through catheter aspiration causes periprocedural hemoglobin drop (4). Some studies have documented that bleeding and hemoglobin loss are associated with cardiac adverse outcomes in patients undergoing PCI and cardiac operation. Kang Hyu et al. (4) have shown that hemoglobin drop (> 1 g/dL) during PCI is an important predicting factor for contrast-induced nephropathy (CIN) that is associated with an increased risk of death and late cardiovascular events after PCI. Ndrepepa et al. (1) have demonstrated a strong relationship between the 30-day frequency of bleeding and 1-year mortality after PCI.

## 2. Objectives

Although percutaneous coronary intervention is an effective treatment of coronary artery disease, it seems necessary to investigate the impact of complications on outcomes including myocardial infarction and possible mortality. The purpose of this study was to evaluate the relationship between periprocedural hemoglobin level and cardiac enzyme changes as a predictor of cardiac adverse outcomes in patients undergoing PCI.

## 3. Patients and Methods

This prospective, descriptive study was conducted on 1012 consecutive patients diagnosed with coronary artery disease who underwent PCI between November 2010 and November 2011 at Rajaie Cardiovascular Medical and Research Center, Tehran University of Medical Sciences. The inclusion criteria were a normal pre-procedural troponin I (TnI) level and creatinine-MB fraction (CK-MB). Patients recently diagnosed with myocardial infarction were excluded. The diagnosis of a pre-procedural myocardial injury was based on either the development of new pathological Q waves in at least two contiguous ECG leads or an elevation of CK-MB > five times the upper limit of normal (ULN) or Tn > 0.06 µu/l. All the patients were treated according to the latest PCI guideline (5), recommending Aspirin, Clopidogrel, and Atorvastatin (low to intermediate doses). According to the peak level of post-procedural CK-MB and TnI, the patients were stratified into four categories: Non significant enzyme rising, patients with CK-MB rising more than three times ULN; those with TnI > 0.06 µu/l and ones with CK-MB rising more than three times ULN and TnI > 0.06 µu/l.

Whole blood hemoglobin was measured via the hematology auto analyzer using a cyanmethemoglobin method. The normal ranges for hemoglobin values were considered as follows: 14-17.5 mg/dl for men and 12-15.3 mg/dl for women (6). In order to distinguish whether there were any hematopathology with respect to hemoglobin amounts, although we considered this term as a continuous variable, participants were classified into anemic (Hb = 10-12) and non-anemic (Hb > 12) groups (7). The samples for TnI and CK-MB were collected before the procedure and at 8, 16, and 24 hours post-procedurally. The samples of TnI were analyzed using the Immune Fluorescent Assay, and the CK-MB levels were measured via an enzymatic method (Biochemical auto analyzer). The ULN for CK-MB and TnI was 25 Iu/l and 0.06 µu/l, respectively. The standard PCI technology was utilized. All patients provided written, informed consent for participation in the study, and the institutional Review Board and the Ethics Committee of Tehran University of Medical Sciences approved the study protocol.

The statistical analysis was performed with PASW Statistics 18 for Windows (SPSS Inc, Chicago, Illinois). The data were expressed as means ± SD for the continuous and as percentages for the discrete variables. The independent samples *t* test or Mann Whitney U test was used to compare the continuous variables between the groups. Pearson's chi-square test (or Fisher's exact test, as appropriate) was performed for the statistical analysis of the nominall variables. A logistic regression model was built including the dependent and independent variables with significance or near significance. Odds ratios and 95% confidence intervals (CI) were presented with two-tailed p values. P values < 0.05 were considered statistically significant.

## 4. Results

Between November 2010 and December 2011, a total of 1126 patients without acute coronary syndrome who underwent elective PCI were enrolled into the present study. Of this total, 111 patients with cardiac enzyme elevation (CK-MB and TnI elevation > five times the ULN) and documented diagnosis of myocardial infarction were excluded. Consequently, 1012 patients (725 men and 287 women) at an average age of 59 ± 10 years were considered for analysis. We should note that with respect to sample analysis for hemoglobin, all of the patients were classified in to the non –anemic group. Also, there were no significant differences in parameters related to medical treatment (Beta blockers, Nitrates, Calcium antagonist, ACE inhibitors, Statins) between groups.

### 4.1. Clinical Characteristics and Hemoglobin Status in Patients According to Post-PCI Cardiac Enzyme Levels

#### 4.1.1. Clinical Characteristics and Hemoglobin Levels of Patients According to Post-PCI CK-MB Status

In this classification cigarette smoking was more prevalent in patients without CK-MB ≥ 3× ULN (P = 0.050). The rest of the clinical characteristics did not differ significantly between the two groups. The biochemical analysis of the blood samples revealed that all of the patients did not have hemoglobin abnormality at baseline (Table 1). However, post procedure hemoglobin levels were less than baseline values. These results were supported by multivariate analysis after adjustment for different independent variables.

**Table 1.** Clinical Characteristics and Hemoglobin Levels of Patients According to Post-PCI CK-MB Status

	CK-MB^a^≥ 3×		P Value
	Yes (n = 46)	No (n = 966)	
**Male Gender**	31(67.4)^b^	694 (71.8)	0.506
**Age (years)**	61.2 ± 11^b^	59 ± 10.9	0.174
**Diabetes**	15 (32.6)	248 (25.7)	0.304
**Hypertension**	19 (41.3)	406 (42.0)	> 0.99
**Hyperlipidemia**	19 (41.3)	395 (40.9)	> 0.99
**Family history of CAD** **^a^**	7 (15.2)	168 (17.4)	0.843
**Current smoking**	8 (17.4)	300 (31.1)	0.050
**Baseline Hb^a^, g/dl**	13.33 ± 1.34	13.30 ± 1.54	0.368
**Postprocedure Hb, g/dl**	12.06 ± 1.47	12.27 ± 1.62	0.368

^a^ Abbreviations: CK-MB, creatinine kinase-MB; CAD, coronary artery disease; Hb, hemoglobin

^b^ Values are expressed as mean ± SD or n (%)

#### 4.1.2. Clinical Characteristics and Hemoglobin Levels of Patients According to Post-PCI TnI Status

In the subjects classified according to TnI status, the positive patients were more likely to be older (P = 0.002). Clinical laboratory tests showed that, although the mean hemoglobin levels decreased post-procedurally, the positive patients had significantly lower hemoglobin levels after the procedure (P = 0.008) (Table 2). But the results of the linear regression model revealed that neither hemoglobin status (1.113 ± 0.879; P=0.257), nor other cardiovascular risk factors (sex;-2.663 ± 2.331; P = 0.255, hypertension; 0.371 ± 2.125; P = 0.858, hyperlipidemia; 0.577 ± 2.119; P = 0.789, family history of CAD; 3.997 ± 2.566; P = 0.120 and smoking; 3.449 ± 2.276; P = 0.129) were exact predictors of post-procedural TnI elevation. 

**Table 2.** Clinical Characteristics and Hemoglobin Levels of Patients According to Post-PCITnI Status

	TnI^a^≥ 5×		P Value
	Yes (n = 546)	No (n = 566)	
**Male Gender**	322	403	0.779
**Age (years)**	60.3 ± 11.3^b^	58.1 ± 10.5	0.002
**Diabetes**	119 (26.7)	144 (25.4)	0.665
**Hypertension**	183 (41.0)	242 (42.8)	0.608
**Hyperlipidemia**	184 (41.3)	230 (40.6)	0.847
**Family history of CAD** **^a^**	68 (15.2)	107 (18.9)	0.132
**Current smoking**	130 (29.1)	178 (31.4)	0.450
**Baseline Hb^a^, g/dl**	13.23 ± 1.56	13.36 ± 1.51	0.180
**Postprocedure Hb, g/dl**	11.51 ± 1.63	12.39 ± 1.60	0.008

^a^ Abbreviations: CAD, coronary artery disease; Hb, hemoglobin; TnI: troponin I

^b^ Values are expressed as mean ± SD or No. (%)

#### 4.1.3. Clinical Characteristics and Hemoglobin Levels of Patients According to Post-PCI CK-MB and TnI Status

Table 3 depicts the baseline clinical characteristics and hemoglobin levels according to both CK-MB and TnI status after procedure. There was no significant difference in the prevalence of cardiovascular risk factors between the positive and the negative groups. Also, Hemoglobin levels at baseline and post-procedurally altered insignificantly. 

**Table 3.** Clinical Characteristics and Hemoglobin Levels of Patients According to Post-CK-MB and TnI Status

	CPK-MB^a^ ≥ 3 and TnI^a^ ≥ 5		P Value
	Yes (n = 176)	No (n = 8366)	
**Male Gender**	115 (65.3)^b^	583(69.7)	0.052
**Age (years)**	59.5 ± 10.55^b^	58.9 ± 11	0.515
**Diabetes**	46 (26.1)	203 (24.3)	0.849
**Hypertension**	67 (38.1)	342 (40.9)	0.273
**Hyperlipidemia**	70 (39.8)	325 (38.9)	0.865
**Family history of CAD** **^a^**	33 (18.8)	137 (16.4)	0.585
**Current smoking**	41 (23.3)	257(30.7)	0.024
**Baseline Hb^a^, g/dl**	13.45 ± 1.34	13.24 ± 1.51	0.325
**Postprocedure Hb, g/dl**	12.51 ± 1.63	12.39 ± 1.60	0.278

^a^ Abbreviations: CK-MB, creatinine kinase-MB; CAD, coronary artery disease; Hb, hemoglobin; TnI: troponin I

^b^ Values are expressed as mean ± SD or n (%)

## 5. Discussion

The main finding of the present study is that post procedure hemoglobin level may be associated with post-PCI cardiac enzyme elevation (particularly troponin I), although this was not supported by the multivariate analysis. This means that hemoglobin dropping can lead to worse clinical outcomes in patients undergoing catheterization procedures. McKechnie and colleagues (8) have proposed that a lower hemoglobin level might be a marker of post-PCI severity and that it could identify patients at higher risk of complications after intervention procedures. Probable mechanism behind this outcome is impaired oxygen delivery to tissues causing hypoxia as well as reduced coronary flow reserve and eventual myocardial ischemia (9). 

Although the samples of our study were in the non-anemic group at baseline, Carson et al (10) have reported that there is a direct relationship between preoperative anemia and coronary bypass surgery mortality and morbidity. Lee and colleagues (7) have demonstrated that moderate-severe (Hb < 10) or mild (Hb < 10 < 12) anemia is associated with increased one-year mortality, hospital length of stay, post-PCI cardiac enzymes, and 30-day MACE. Anemic patients undergoing PCI are liable to higher oxygen demand and at risk of exacerbated supply disturbance during the procedure. This causes an increase in cardiac enzymes after PCI probably due to less well-tolerated ischemia induced by balloon inflation during the procedure (11). Another probable mechanism is that mobilizing bone marrow stem cells in the circulation moving toward myocardial infracted site can regenerate de novo myocardium and coronary blood vessels (12, 13). This may be impaired in the presence of anemia (7). 

Although in our study age was an independent predictor of cardiac enzyme increase, the relationship between hemoglobin levels and gender in adverse outcomes are controversial. Previously in the Framingham study, it was observed that women with lower hematocrite values were at the risk of cardiovascular events more than men (14). McKechnie and colegous (8) supposed an increased post-procedural myocardial infarction in women. Kang Hyu et al (4) have revealed that there was a direct relationship between lower hematocrit level and risk of cardiovascular death, particularly in men. 

There are some independent periprocedural predictors including antiplatelet agent treatment, increased procedural duration, increased time to sheath removal, length of intensive care unit stay and use of an intra-aortic balloon pump that are involved in major hemorrhages following PCI (15) although another report has noted a marked decline in the incidence of major femoral bleeding after PCI over the past decade (16). In all categories classified by enzyme status, positive patients were older. Older age was an independent predictor for post procedural troponin I increase (p = 0.002). McKechnie et al. in their cohort study on patients who had undergone PCI have suggested that anemic men and women were older and had a higher percentage of comorbidities after procedures (8). Post-procedure hemoglobin dropping may be considered as a predictor of cardiac adverse outcomes in patients undergoing PCI although this theory was not supported by logistic regression. We suggest that a good bleeding control during and after the procedure can reduce the risk of cardiac enzyme elevation post-procedurally.
